# Research progress on the mechanisms of pain empathy

**DOI:** 10.1002/ibra.12169

**Published:** 2024-06-30

**Authors:** Shuangshuang Liu, Siwei Wang, Yan Yan, Bangyong Qin, Qingxiang Mao, Jie Yuan

**Affiliations:** ^1^ Department of Anesthesiology/Department of Pain Medicine Affiliated Hospital of Zunyi Medical University Zunyi China; ^2^ Department of Anesthesiology, Daping Hospital Army Medical University Chongqing China; ^3^ Guizhou Key Laboratory of Anesthesia and Organ Protection Zunyi Medical University Zunyi China; ^4^ Department of Oral Implantology Hospital of Stomatology Zunyi Medical University Zunyi China; ^5^ Department of Surgery and Cancer, Nociception Group, Division of Anaesthetics, Pain Medicine and Intensive Care, Chelsea and Westminster Hospital Campus Imperial College London London UK

**Keywords:** empathic response, mirror neurons, neural mechanisms, pain, pain empathy

## Abstract

Recent research has highlighted the indispensability of traditional molecular biology and imaging techniques in pain research. However, the mechanisms underlying pain empathy remain unclear. Consequently, a deeper understanding of these mechanisms would greatly enhance pain management. This article aimed to scrutinize previous research findings on pain empathy, with a particular emphasis on the correlation between empathy for pain and distinct anatomical structures, such as mirror neurons, the anterior cingulate cortex, insular cortex, prefrontal cortex, and amygdala. Additionally, this study explored the involvement of endogenous systems, including oxytocin and the locus coeruleus norepinephrine system, hypothalamic–pituitary–adrenal axis, opioid system, and 5‐hydroxylamine signaling. In conclusion, the mechanisms of pain empathy are complex and diverse, and research on pain empathy and target treatment will contribute to pain treatment.

## Graphical abstract

This article is related to the graphical research progress of pain empathy, starting with the description of the pain matrix and mirror neurons. Based on the pain matrix and mirror neurons, pain empathy results from the combined action of brain regions such as the anterior cingulate cortex, insula, prefrontal cortex, and amygdala and regulatory substances such as oxytocin, the locus coeruleus–norepinephrine system, hypothalamic–pituitary–adrenal axis, opioid system, and 5‐hydroxytryptamine receptor. The goal of this article is to better understand the mechanisms of pain.

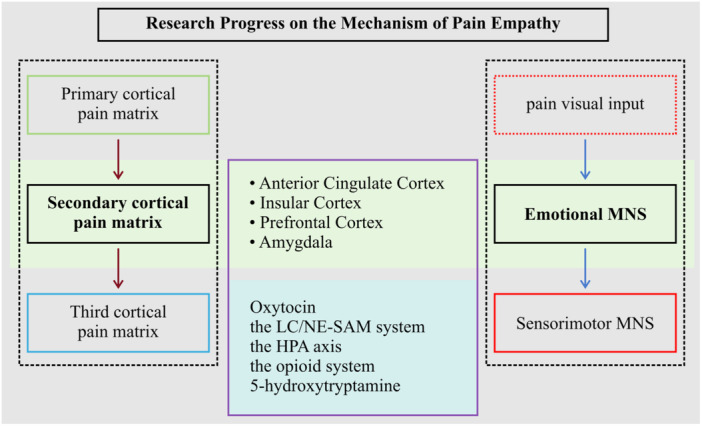

## 1 INTRODUCTION

Empathy encompasses three dimensions: cognitive, affective, and behavioral. Cognitive empathy involves the capacity to identify and comprehend another individual's experiences as well as effectively communicate and validate this understanding with the person in question. It is often described as perceiving the world from another person's perspective. Affective empathy centers on recognizing and identifying with another person's emotional state and is characterized by emotional resonance with the individual. According to a model of empathetic behavior, the affective state of a conspecific can automatically activate the nervous system to map the affective states of others onto our own emotional state.^1^ Research provides evidence that empathy for pain can alter how humans experience pain through social mediation, leading to hyperalgesia or paresthesia.^2^ Richardson et al. found that children develop brain functional differentiation by the age of 3 to understand other people's bodily feelings (usually pain experiences). Regarding the negative psychological states of others (emotions, ideas, etc.), further evidence exists that differences in somatic and emotional processing are already developing early in human development when dealing with others' pain.^3^

The study of empathy for suffering has a great deal of social significance as well as significant research value for human survival and development. On one hand, pain empathy can help individuals comprehend the suffering of others and act appropriately, leading to more prosocial behavior. On the other hand, the perception of others' suffering can also serve as a warning signal for hazardous situations, reminding individuals to prepare for defense. Research on pain empathy has a long history and understanding its mechanisms can help in the treatment of pain. Scholars have posited that pain empathy is rooted in personal suffering; however, few studies have focused on empathy for pain from the perspective of neurophysiology. This study reviewed the literature on pain empathy, highlighting its neural mechanisms and exploring the potential for pain treatment.

## 2 RESEARCH HISTORY

Owing to its high stability, reproducibility, and efficacy, the rat model of pain empathy is widely used for investigating the neural mechanisms of empathy. Some of the earliest evidence for emotional cognition is found, in which, rats (observers), trained to push levers to get food rewards, decreased the force to press the levers when they saw another rat (a demonstrator) receiving foot shocks. This research examined how demonstrator and observer rats transmitted their emotional states to one another.^4^ Rats and mice also showed evidence of social transmission of analgesia and pain.^5^, ^6^ Langford et al.^7^ used the mouse grimace scale in 2010 to identify three specific expressions of facial pain: eye closure, cheek puffing, and nose puffing. Notably, they found that mice could communicate pain with these expressions to seek help from peers. In research by the Mogil laboratory,^8^ a pair of mice was placed in an observation box where they could communicate directly, and then, acetic acid was injected intraperitoneally. It showed that the pair's pain threshold decreased in tandem, and acetic acid‐induced writhing increased in its cage mates, although no such response was observed in mice that were not housed in the same cage. In 2014, researchers devised a study in which a rat, transferred to a different observation box after 30 min of socialization with pain‐injured rats in the same cage, exhibited the decreased pain threshold and increased pain response when treated with subcutaneous injection bee venom. However, no such response occurred between unfamiliar rats housed in different cages. Furthermore, the researchers identified that empathy for pain in rodents is dependent on familiarity and the medial prefrontal cortex is essential for processing the enhanced spinal cord nociception that is linked to empathy.^9^

Similarly, researchers have studied human empathy extensively. In 2004, Singer et al.^10^ examined differences in the brain networks activated by receiving pain stimulation oneself (direct feeling) and watching a spouse receive pain stimulation (indirect feeling). They found that the brain areas responsible for the empathy of pain, including the anterior cingulate cortex (ACC), bilateral anterior insula (AI), brainstem, and cerebellum, were specifically activated when witnessing the pain of a spouse. When participants were presented with in‐person pain stimuli, the somatosensory cortex, somatic motor cortex (M1), and affective cortex were all found to be activated. Corradi‐dell'Acqua et al.^11^ found that the AI and ACC are crucial for management of pain and aversive sensations. In some meta‐analyses,^12^, ^13^, ^14^ the insula, anterior cingulate gyrus, and prefrontal cortex (PFC) were confirmed to be activated by the nucleus during empathy for pain. However, other regions of activation include the posterior central gyrus, inferior parietal lobe(IPL), thalamus, brainstem, bilateral inferior frontal gyrus(IFG), and cerebellum. These results provide evidence of major neurochemical regulation systems in pain empathy, suggesting that pain empathy can be studied from multiple perspectives, including neural anatomical structures and neurochemical regulation (Table 1).

**Table 1:** Recent research on empathy mechanisms.

Region	Year	Major finding	Research object	Reference
Anterior Cingulate Cortex	2018	Emotional changes may cause aberrant augmentation of synaptic transmission in the ACC, increasing dorsal horn neuron activity via the top‐down route. Potentiated spontaneous and stimulation‐induced activity in spinal cord neurons will provide a pseudo “pain”‐like feeling in the absence of unpleasant peripheral inputs.	Rat	[15]
	2019	Mirror neurons with enhanced activity after viewing the pain of other animals have been found in the rat ACC.	Rat	[16]
	2021	The dorsal raphe nucleus‐ACC 5‐hydroxytryptaminergic neural circuit is associated with comfort like behavior and social skills.	Voles	[17]
	2021	After fast empathetic pain training, the ACC‐nucleus accumbens pathway is engaged. In mice, optogenetic suppression of the ACC‐nucleus accumbens circuit reduces behavioral empathetic suffering.	Mice	[18]
Insular Cortex	2020	By activating cortical OTR and engaging GABAergic transmission, OXT at the rostral agranular insular cortex level can block nociceptive input at the spinal cord level, increasing spinal α2A‐adrenoceptor activity.	Rat	[19]
	2021	Intra‐ACC or INS injections of CoCl_2_ abolished the anxiogenic effect in mice living with a chronically ill conspecific.	Mice	[20]
	2021	The anterior insula and brain areas associated with sensory discrimination were not only shared but also worked as a mediator between pain severity and pain empathy in primary dysmenorrhea patients.	Human	[21]
	2022	Glutamatergic projection from the insular cortex to the basolateral amygdala is essential for pain perception. The ACC is more likely to be engaged in the development/induction of empathic distress, whereas the INS may be important in forming and consolidating empathic distress.	Mice	[22]
Prefrontal Cortex	2011	In addition to excitatory glutamate, the cholinergic signaling pathway through nicotinic receptors in PFC promotes the exploration of new social stimuli.	Mice	[23]
	2021	Masculinity, and not femininity, was associated with an increase in the mPFC's empathic response to high‐pitched infant sounds but not low‐pitched ones.	Human	[24]
Amygdala	2015	Activity in the amygdala was correlated with empathic regulation of the emotional suffering of others, rather than physical pain.	Human	[25]
	2021	Serotonergic signaling from the amygdala contributes to empathy‐like behavior.	Mice	[26]
	2023	The involvement of 5‐HT3R and GABAergic mechanisms within the amygdala contributes to empathy for pain model‐induced pain hypersensitivity.	Mice	[27]

Abbreviations: 5‐HT3R, 5‐hydroxytryptamine receptor; ACC, anterior cingulate cortex; GABA, gamma‐aminobutyric acid; INS, insular cortex; OTR, oxytocin receptor; OXT, oxytocin; PFC, prefrontal cortex.

## 3 NEURAL MECHANISMS OF PAIN EMPATHY

### 3.1 Pain matrix

**Figure 1:**
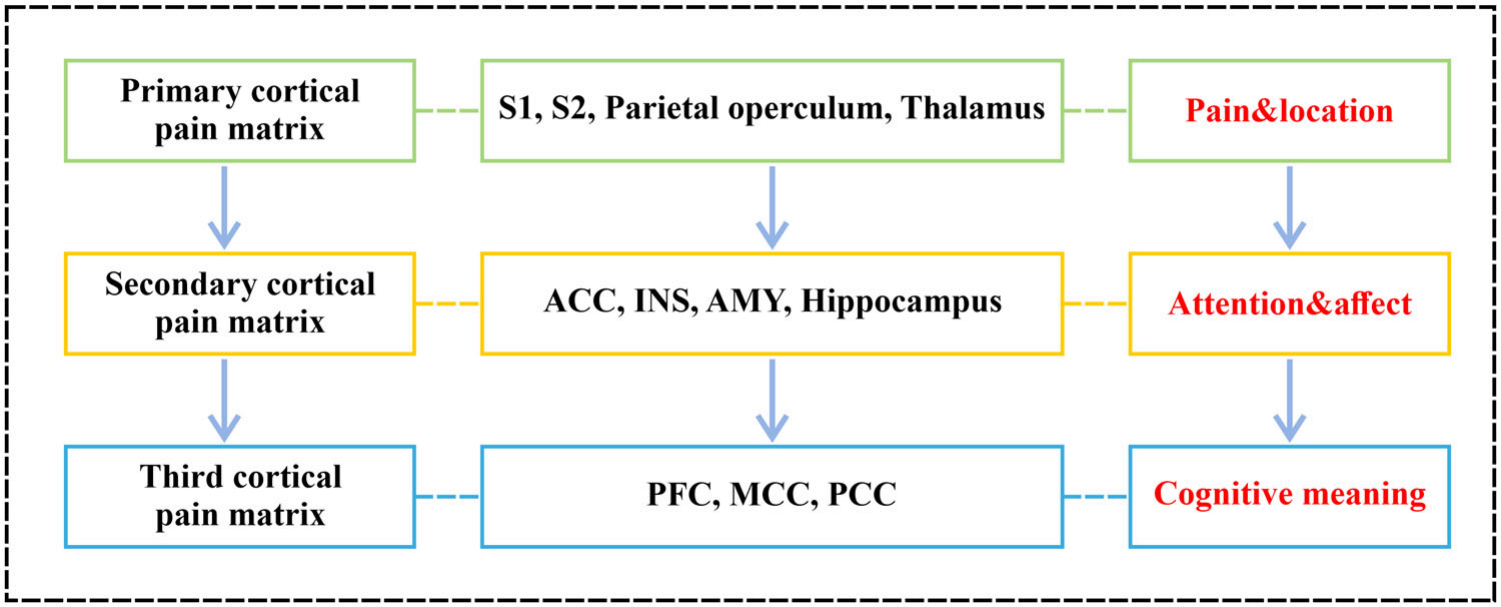
Three tiers are present within the pain matrix. Perception and localization of pain are influenced by the primary cortical pain matrix, which consists of the S1, S2, parietal operculum, and thalamus; the ACC, INS, AMY, and hippocampus comprise the secondary cortical pain matrix, which is responsible for empathy for suffering; the third region, which includes the PFC, MCC, and PCC, is linked to cognition. ACC, anterior cingulate cortex; AMY, amygdala; INS, insula; MCC, medial cingulate cortex; PCC, posterior cingulate cortex; PFC, prefrontal cortex; S1, primary somatosensory cortex; S2, secondary somatosensory cortex. [Color figure can be viewed at wileyonlinelibrary.com]

The shared pain network between pain perception and alternative pain experiences includes part of the pain matrix, which is the neural network responsible for experiencing pain. This network includes components of emotional motivation, including the ACC, midcingulate cortices (MCC), AI, and primary (S1) and secondary somatosensory cortices (S2) (Figure 1).^28^ Pain perception and location are influenced by the primary cortical pain matrix, which comprises the posterior insula, the parietal operculum, and S1 and S2. Empathic suffering is linked to the amygdala, ACC, hippocampus, and AI secondary brain areas. The third region is associated with cognition and consists of the medial and posterior cingulate cortices as well as the frontal cortex.^29^ The primary way to investigate empathic experiences in the brain involves examining changes in activity within specific regions of the pain matrix. The pain matrix is usually divided into two conceptually different subdivisions, namely, the emotional motivation subdivisions (emotional quality of preparing for pain emotions) and the sensory discrimination subdivisions (dealing with the sensory aspects of pain).^30^ When observing other individuals in pain, the emotional/motivational regions of the pain matrix (especially the cingulate cortex and AI) continue to be activated.^31^ Some people believe that the sensory/discrimination area of the pain matrix can only be activated when the injured area is observed, rather than when only suggesting pain.^31^ Brain stimulation studies have also shown that when directly observing pain, the empathy of pain can be simulated. These findings indicate that pain stimuli and pain‐related empathy responses only share a portion of the pain matrix. Sympathizing with the pain of others primarily involves emotional ACC‐ and insula‐mediated processes. Previous studies have reported interactions between different subunits of the pain matrix during the direct pain experience. For example, Valet et al.^32^ found that the ACC exerts a top‐down influence on the midbrain periaqueductal gray matter and the posterior thalamus to regulate pain during distraction. The downward impact of the ACC may inhibit the transmission of nociceptive sensations through brainstem structures.^33^ The ACC is connected to most of the sensory and somatosensory cortices. Considering that functional connections between these brain regions also increased during directly and psychologically induced pain, we propose that the ACC can regulate somatosensory activity through direct connections. Because of the crucial role of the ACC in descending pain regulation, ACC activation induced by perceived pain may first regulate subcortical structures such as the lateral thalamic nucleus, which in turn regulates S2 activity.^34^ For individuals with poorer economic conditions, the empathetic responses of the anterior midcingulate cortices (aMCC), insular postcentral gyrus, and temporoparietal junction increase, indicating that economic conditions can regulate empathetic responses in the pain matrix.^35^ The empathetic neural response of the pain matrix also varies by the target's race based on within‐group/‐outgroup relationships. Functional magnetic resonance imaging (fMRI) has shown that in Caucasians and Chinese people, pain stimuli applied to faces of the same race lead to increased activation of the ACC and the subfrontal/insular cortex. However, when the participants saw faces from other races, the empathy response of the ACC was significantly reduced.^36^

A magnetoencephalography (MEG) study on empathy for pain revealed that imagination–anticipation of pain in others is mapped in the primary somatic cortex, as indicated by suppression of 10 Hz oscillations. Therefore, MEG may be the most effective method for studying fast‐occurring interarea crosstalk.^37^ The increase in pain observation‐related γ‐band synchronization is associated with robust anatomical and functional connections among primary somatosensory motor cortex neurons.^38^ A lack of γ‐band sensorimotor coherence increase during pain observation may be a sensitive sign of empathic responsiveness in many clinical circumstances.^39^

In several preliminary experiments, researchers found a strong correlation between the modification of event‐related potentials (ERP) and empathic pain. Regarding the standard ERP elements in pain empathy research, among the psychophysiological neural markers of pain empathy, four components are usually revealed in the comparison of pain and non‐pain pictures: N1 and N2 components reflect the early automatic emotion‐sharing process, while P3 and LPP components reflect the late top‐down controlled cognitive evaluation process. In one study, the participant performed a behavioral test while electroencephalographic (EEG) activity was monitored. The researchers collected the peak amplitudes of the ERP components in response to empathetic stimulation of painful faces or hand stimuli, as well as neutral images. In the electrophysiological findings, the P2, N170, N2, and P3 ERP components were associated with modulation of empathetic emotions.^40^ In psychophysiological research, ERP components that occur between 100 and 300 ms are linked to affective sharing and perception processes and are not affected by the cognitive assessment process.^41^ However, the latter components (more than 300 ms) are linked to the identification of emotions and the development of cognitive‐evaluative methods.^42^

### 3.2 Mirror neurons

**Figure 2:**
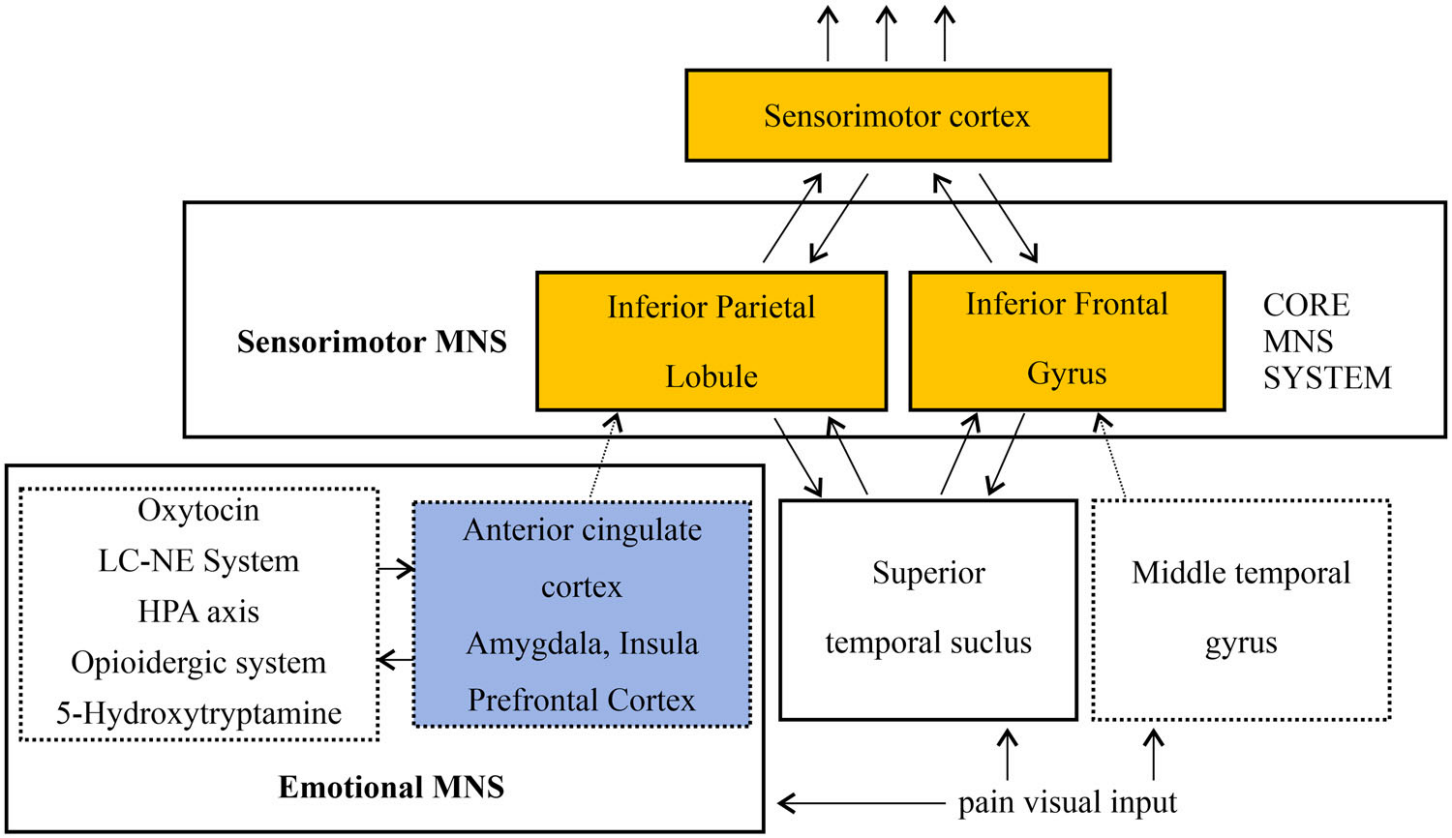
Schematic of areas in the human brain that contain mirror neurons (inferior parietal lobule and inferior frontal gyrus) and make up the “core” system. The “extended” mirror neuron system (anatomical and functional circuits) involves additional brain areas, e.g., anterior cingulate cortex, amygdala, insula, prefrontal cortex, superior temporal sulcus, and middle temporal gyrus; functional perspectives propose that one critical component of the “extended” mirror neuron system is the sensorimotor cortex. Oxytocin, the LC/NE system, the HPA axis, the opioid system, and 5‐hydroxytryptamine are regulators in the “extended” mirror neuron system. The “extended” mirror neuron system connects to the core system and performs transformations on the data critical for mirroring and pain simulation. HPA, hypothalamus–pituitary–adrenal; LC/NE, locus coeruleus–norepinephrine; MNS, mirror neuron system. [Color figure can be viewed at wileyonlinelibrary.com]

It is reported that multimodal information concerning others, such as actions, emotions, feelings, and communication information, is mapped onto the observer's neural base and processed through mirror neurons.^43^ Following the discovery of mirror neurons, it became of primary interest to neuroscientists to study empathy. The presence of mirror neurons indicates that action and perception are governed by the same neural sublayer, and this network has developed to identify the emotions of others. Some researchers propose that common emotional feelings can be traced back to the mirror neuron mechanism. The mirror neuron system (MNS) was first proposed in 1992,^44^ which is known as the core system responding similarly to observed and executed actions. Mirror neurons are located in the PFC and IPL, which were originally discovered in the brains of nonhuman primates, such as macaques. Combining literature reports,^45^, ^46^ the MNS may be related to the processing of observed pain. Mirror neurons mainly exist in core MNS areas such as the IPL and IFG. The “extended” MNS (anatomical and functional circuits) involves additional brain areas, for example, the ACC, amygdala, Insula, PFC, superior temporal sulcus, and middle temporal gyrus. The MNS is divided into the sensorimotor and emotional MNS. The sensorimotor MNS is mainly involved in motor function and consists of the core areas of the bilateral IFG and IPL. The emotional MNS plays a role in the expression, experience, and perception of emotional expressions on the face and body. This network includes regions such as the ACC, amygdala, and insula; among them, some regions themselves may not contain mirror neurons, such as the superior temporal sulcus. These brain regions connect to the core system and transform data critical for mirroring and pain simulation. However, physical–motor processing also occurs during emotional processing, indicating that the sensorimotor MNS may also be activated when experiencing empathy. In addition, subcortical connections support the interaction between these two MNS components. The ACC and amygdala are structurally connected to the premotor cortex (including the IFG), whereas the insula has extensive connections to the premotor cortex and IPL (Figure 2). fMRI is often used to learn about empathy in pain since it can reveal activated brain nuclei and relative changes both in real time and synchronously, which allows for a more precise exploration of the neural mechanisms of empathy for pain. By using fMRI, a groundbreaking study compared brain recruitment regions, in which volunteers were undergoing painful stimulation or watching a loved one undergoing the same stimulation. It was found that the joint MNS was activated in the IFG, parietal cortex, fusiform gyrus, posterior superior temporal sulcus, and amygdala. By a combination of near‐infrared spectroscopy, positron emission tomography (PET), transcranial magnetic stimulation, electroencephalography, electromyography, magnetoencephalography, and MRI, it was shown that a large amount of neural activity of mirror neurons and the observed excitation can reflect other neurons.^47^ In one study using the rat model of empathy, Carrillo et al. employed single‐cell recordings and pharmaceutical intervention to induce a “lesion.” After being trained on observations of similar suffering, the authors demonstrated that a multivariate decoder could be used to predict empathy for pain.^16^ Intriguing evidence comes from studies showing that analgesics, which alter pain neurons, also alter how humans and rodents respond to the pain of others through a variety of empathy‐related phenomena.^18^ These results provide further neurophysiological support for the link between the MNS and empathy for pain.

Human brain imaging and rodent electrophysiological data have demonstrated empathy‐related neuronal activity in the ACC and insula_._ Previous research has suggested that the ACC and insula have cognitive components in empathic pain and emotional information processing, respectively.^48^ Furthermore, pharmacological inhibition of the insula prevented chronic pain hyperalgesia generated by mouse cohabitation, indicating altered emotional contagion.^49^ In a single investigation, the mirror and anti‐mirror characteristics of ACC and insula neurons were analyzed. The activation patterns for the mirror and anti‐mirror combinations were comparable, peaking before the socially active responses associated with empathy, including nostril interactions, prosocial assistance, and social approaches. The insula maintains a balanced ratio between mirror and anti‐mirror neurons, whereas the ACC has a higher proportion of mirror neurons because its linked tissue comes from pain transmission in both oneself and others. These groups of neurons are organized into a brain circuit.^48^ In individuals with highly psychopathological features, as well as in areas related to emotional processing and transfer, mirror neurons exhibit low activation. These results suggest that the mirror nervous system of psychiatric patients undergoes changes that exacerbate obstacles to emotional transfer.^50^

Currently, three widely employed techniques are available for quantifying the activity of mirror neurons. Initially, fMRI was used to evaluate variations in blood‐oxygenation level‐dependent responses in regions of the MNS during action observation, with high spatial resolution.^51^ EEG is the second method for measuring mirror neuron activation in a high‐resolution fashion. An alternative approach to potentially quantify the activation of mirror neurons with high temporal resolution is using EEG mu‐suppression. Large‐amplitude EEG oscillations in the mu frequency band (8–13 Hz) are commonly observed over the scalp regions Central Zero, Central 3, and Central 4 or Frontal Pole 3 and Frontal Pole 4 (the electrode placement positions of EEG) when motor neurons are at rest. These oscillations result from the spontaneous and synchronous firing of sensorimotor neurons.^52^, ^53^ Furthermore, mirror neurons can be evaluated by observing alterations in corticospinal excitability when M1 is subjected to single‐pulse transcranial magnetic stimulation (TMS). TMS is a noninvasive neuromodulation technique in which magnetic pulses traverse the skull to induce electrical currents in the brain.^54^ While changes in neural activity, as assessed through methods such as mu‐suppression, corticospinal excitability, or blood‐oxygenation level‐dependent response, can be utilized to deduce the activation of mirror neurons, it is essential to acknowledge that neural activation observed may not exclusively be indicative of mirror neurons. Such measurements reflect mass neural activation rather than the more precise single‐cell recordings commonly employed in animal studies.^55^

### 3.3 ACC

A meta‐analysis^13^ of neuroimaging studies has shown that the cingulate cortex is the most active area of the brain involved in cognitive empathy and empathy for pain. Studies of evoked potentials in humans have revealed that the ACC is vital for processing pain information. The anterior cingulate area A24 mirror emotion neurons are stimulated by personal pain experience and the observation of pain in another individual.^56^ As is well known, the ACC can regulate the emotional motivational components of pain and lead to overactive neuronal responses to pain. Studies showed that ACC neurons exhibit synaptic plasticity under chronic pain conditions.^57^ In humans and rodents, the ACC is particularly important for emotional and motivational responses to direct and observed pain, as well as social transmission of pain, mediating different forms of empathy by influencing different downstream targets. The ACC is believed to communicate with a wide range of brain regions that regulate emotional and motivational states, including the thalamus, insula, amygdala, and nucleus accumbens.^18^ Furthermore, according to one report, the expression of activity‐regulated cytoskeleton‐associated protein (ARC) is heightened in neurons of the rodent ACC after directly experiencing or observing another animal undergoing foot shocks.^58^ Immunohistochemical results showed that cohabitation with a pair of mice under chronic pain conditions resulted in a bilateral decrease in the activation pattern of FosB markers in the mouse ACC, which may be related to the activation of local GABAergic neurons.^20^ Xiao and Zhang^59^ reported that inhibition of its neural activity removed negative emotions related to neuropathic, inflammatory, and visceral pain in an animal model with lesions of the dorsal anterior cingulate neural layer. Furthermore, mechanical hypersensitivity in observer mice was reduced by optogenetic deletion of the anterior cingulate area A24 neurons stimulated by interaction with a conspecific in the presence of pain.^18^, ^56^ In optogenetic studies, selective activation of ACC–spinal projection neurons contributed to the induction of sensitization of nociceptive responses. Although their inhibition relieves neuropathic pain, the ACC directly increases sensory transmission in the spinal cord, and its promotion from the top down contributes to chronic neuropathic pain.^15^ Through in vivo electrophysiological techniques, researchers found that the firing activity of neurons in the ACC of bystander mice increased significantly on the first day of training and gradually weakened or even disappeared after 7 days. Continuous enhancement of neuronal discharge activity in the insula region from Days 1 to 7 of the training period indicated that the ACC was involved in the building of sympathetic pain behavior, whereas the insula was involved in the formation and consolidation of sympathetic pain behavior. Synaptotagmin‐2 and Rab3‐interacting molecule‐3 are required for enhanced glutamate synaptic transmission from the right insular cortex to the proper basal lateral amygdala circuit.^22^ In conclusion, the ACC is a crucial brain area that regulates empathy behavior and neuronal firing activity in collaboration with other brain regions, thereby promoting pain sensitization.

### 3.4 Insular cortex

The insula is essential for regulating nociception and is associated with pain behavior, in which, many neurons are connected to different brain regions involved in pain‐related emotional characteristics.^60^ During the 1990s, the rapid development of imaging techniques facilitated the investigation of the function of the insula. Hsieh et al.^61^ used PET to show that bilateral anterior islets and other related areas of the brain increase regional cerebral blood flow during pain. Lu and colleagues^62^ demonstrated that the insula shows strong activation in the dynamic time course of the affective state (e.g., depression and anxiety) of pain. Increased FosB expression in the insula is linked to activation of glutamatergic neurons, and excessive arousal in this region may increase anxiety‐like responses. A local injection of cobalt chloride was found to reduce glutamate neurotransmission via chemical inhibition of the insula, resulting in anxiety reduction.^20^ Oxytocin is an integral component of empathy and pain. When oxytocin has been microinjected into the insula of rats, it may reduce the pain behaviors and spontaneous discharges of spinal neurons caused by the injection of formalin into the hind legs.^19^ Gamma‐aminobutyric acid (GABA) is the principal inhibitory neurotransmitter in the mammalian brain. Structures containing GABAergic receptors modulate emotional and pain processes. Multiple studies show that the insula of both humans and rodents plays a crucial role in various forms of empathetic behavior, from social regulation of pain to helping behavior. In rodents, allogeneic transplantation of stress‐induced allogeneities increased the expression of c‐Fos in oxytocin receptors (OTR) expressing neurons in the insula of mice,^63^ and inactivation of the insula prevented socially induced hyperalgesia in mice.^49^ With systemic or insula injection of the GABA receptor agonist midazolam, scholars observed that high‐dose benzodiazepine could reverse empathy‐induced hyperalgesia, indicating that the neurotransmitter system may be involved in affective processing and empathy related to pain.^49^ Cohabiting with a mouse in chronic pain increased the nociceptive response in mice living in pairs, but turning off the insula reversed this reaction. Systemic midazolam reduced pain, but intrainsular midazolam had no effect. The insula may control this reaction, and the GABA system may be involved but not its insular mechanism_._ ^49^ In conclusion, pain empathy is closely related to the insula, which mainly promotes pain empathy through oxytocin or GABAergic receptors.

### 3.5 PFC

The PFC consists of the caudal PFC, ventrolateral PFC, dorsolateral PFC (DLPFC), medial prefrontal cortex (mPFC), and orbitofrontal cortex. Following damage to the bilateral mPFC of rats, there was no difference in the number of painful foot reflexes elicited by the injection of bee venom between observation rats and normal rats, indicating that the mPFC is a significant brain region mediating empathy for pain in rats.^9^ The mPFC is the brain area where ethanol operates by activating GABAA receptors to cause pain empathy‐like behavioral alterations. The similarities in GABAA receptor activation in the mPFC by ethanol and oxytocin imply that they have the same structure and neural pathways that produce emotional empathy for pain and other prosocial and altruistic behaviors, which require further investigation.^64^ Research on a mouse model of autism has shown that multiple pharmacological targets of the mPFC‐basolateral amygdala (BLA) circuit are associated with dysfunctional neural regulation. Research has shown that the DLPFC region is significantly involved in the cognitive components of empathy response, such as emotional regulation and viewpoint adoption.^65^, ^66^ Transcranial direct current stimulation (tDCS) of the DLPFC, right temporoparietal junction, and ventromedial prefrontal cortex can regulate empathy and is a potential tool for treating diseases with empathy disorders.^67^ Subjects who received anodal tDCS of the DLPFC also showed increased ratings of others' pain, consistent with the function of the PFC in empathy for pain. Wang et al.^68^ found that tDCS of the PFC improved self‐rated empathy for pain in participants when they viewed images of patients undergoing painful procedures. In a study using real‐time fMRI to measure the response in the left DLPFC to images of bitter experiences from both the “self” and the “other” point of view, the results showed that the DLPFC participates in the subjects' imagination of other people's pain and adjusts brain activity in the region. Understanding the role of the DLPFC in empathy is critical because its location is appropriate for using neuromodulation techniques, such as tDCS.^28^ The development of new autism treatment agents through local or systemic administration provides valuable clues. In the future, treatment based on neural circuits can be used to treat social dysfunction.^69^ As mentioned above, the PFC has become a therapeutic target for empathy disorders, mainly regulating pain empathy through oxytocin receptors or GABAA receptors and the mPFC‐BLA circuit.

### 3.6 Amygdala

The amygdala is involved in fear and conditioning and is linked to the PFC.^70^ Pain patients exhibit heightened functional connectivity between the left amygdala and various cortical, subcortical, and cerebellar regions. Research has found that oral administration of acetaminophen in rats reduced levels of oxytocin and vasopressin in the amygdala, thereby alleviating the empathetic response. One study examined the activation and inhibition of the 5‐hydroxytryptamine receptor (5‐HT3R) systemically and intra‐amygdally in rodents that cohabited with a conspecific who had sustained a protracted constriction injury. The results suggested that amygdala serotonergic signaling via 5‐HT3R is involved in empathy‐like behavior.^26^ Furthermore, the 5‐HT3R and GABAergic mechanisms within the amygdala are involved in pain hypersensitivity induced by the empathy for pain model. Additionally, they proposed that midazolam and cannabidiol may serve as novel therapeutic options for relieving emotional pain disorders.^27^ One study investigated the neural circuits responsible for deliberately modulating empathic responses to the agony and suffering of others. It revealed that amygdala activity was associated with empathic regulation of others' emotional pain but not their physical pain.^25^ A single high‐dose or repeated low‐dose oral administration of acetaminophen reduced empathy‐like behavior in rats due to a reduction in oxytocin in the PFC and amygdala.^71^ Acetaminophen reduces sympathetic behavior in rats in a dose–response relationship (single high dose and increasing dose), which is related to oxytocin and vasopressin, as well as endogenous cannabinoid systems in the amygdala and prefrontal cortex.^71^ In conclusion, the insula is also an important part of pain empathy research, and its main regulatory substances include oxytocin, vasopressin, 5‐HT3R, and GABAA receptors.

## 4 REGULATORS OF PAIN EMPATHY

### 4.1 Oxytocin

Oxytocin is also thought to provide a fundamental biological basis for empathy,^72^, ^73^ since increasing evidence suggests that the neuropeptide oxytocin enhances empathy (Table 2). In the context of pain processing, listening to a mother's speech can reduce preterm infants' pain scores and increase their saliva oxytocin levels.^74^ The large paraventricular nucleus (PVN) cell neurons, the supraoptic nucleus of the hypothalamus, and the paranucleus are the sites where oxytocin is generated in the vertebrate brain.^75^ The projection of oxytocin neurons is widely distributed in the regions responsible for cognitive function_._ ^76^ In addition, large‐cell neurons project widely to numerous forebrain regions, including the PFC, hippocampus, medial and central amygdala, anterior olfactory nucleus, nucleus accumbens, and lateral septum.^77^ The relationship between oxytocin and cortisol is pain‐related, and the former may inhibit the hypothalamic–pituitary–adrenal (HPA) axis functions at multiple levels during cortisol production.^78^ Research shows that empathy can increase peripheral levels of oxytocin in healthy individuals.^79^ Previous studies have shown that oxytocin can reduce first‐hand pain sensitivity, while recent studies have shown that intranasal administration of oxytocin can indirectly reduce pain empathy scores by reducing first‐hand pain sensitivity.^80^ The amygdala's central nucleus (CeA) regulates anxiety, learning, reinforcement, and memory. The CeA is rich in oxytocin receptors, and oxytocin has been shown to have dose‐dependent, positive potentiating, and anxiolytic effects in the rat CeA. One study found an association between intranasal oxytocin and empathy in facial pain in one ethnic group but not in other races.^72^ For humans, researchers analyzed the amygdala, ACC, and AI in the form of an empathic network. They tested the single‐nucleotide polymorphism rs53576 in the gene encoding the OTR, and they expected greater oxytocin activation in the network area during empathy.^81^ In addition, they hypothesized that risk alleles (AA and GA) would show altered activation in empathy‐related networks following oxytocin release. Furthermore, this result indicates that individuals with the G/G genotype of the oxytocin receptor gene (rs53576) had neural activity that was more sensitive and flexible in empathizing with social relationships than those with the A/A genotype.^82^ Future studies that directly manipulate the oxytocin system need to be conducted to explore the mechanism of pain empathy.

**Table 2:** Regulation of pain empathy.

Brain region	Regulators	Neural mechanisms
ACC	GABA system, opioid system	ACC can regulate the emotional and motivational components of pain and lead to overactive neuronal responses to pain.
INS	Oxytocin, GABA system, opioid system	INS participates in pain‐related emotional processing and empathy through the neurotransmitter system.
PFC	Oxytocin, GABA system, LC/NE system	The PFC region is significantly involved in the cognitive components of empathy response.
AMY	Oxytocin, 5‐HT_3_, GABA system, HPA axis	Amygdala activity was associated with empathic regulation of others' emotional pain but not their physical pain.

Abbreviations: 5‐HT3, 5‐hydroxytryptamine 3; ACC, anterior cingulate cortex; AMY, amygdala; GABA, gamma‐aminobutyric acid; HPA, hypothalamus–pituitary–adrenal; INS, insula; LC/NE, locus coeruleus–norepinephrine; PFC, prefrontal cortex.

### 4.2 Locus coeruleus norepinephrine system

The locus coeruleus (LC) projection structure is related to emotion regulation in the ACC, which shows excessive activity in patients with depression, indicating that the LC has chronic pain‐induced anxiety‐ and depression‐like consequences.^83^ In addition, a bilateral increase of norepinephrine (NE) in the PFC is associated with long‐term pain.^84^ The LC, which receives signal input from numerous brain regions and projects signals to neuronal centers, including the forebrain, cerebellum, brainstem, and spinal cord, is primarily responsible for NE synthesis. LC neurons regulate wakefulness by releasing NE and are involved in learning behaviors.^85^ Sprague–Dawley rats were housed in the same cage as observation mice for 30 min with the usage of a microinjector to deliver bee venom to the soles of CO rats (a naive cagemate observer; CO rats refer to a group of rats that have been raised with pain rats for more than 2 weeks and interacted with pain rats for 30 min before the experiment began). At the same time, videos were recorded, and Von Frey monofilaments were used to assess the extent to which the rats were sensitive to pain. In this study, they condensed in‐depth advice on modeling empathy for pain.^86^ Social interactions with familiar and pained peers resulted in hyperactivation of the LC/NE‐SAM system. Sympathetic‐postganglionic NE increases P2X3 receptor and α1 adrenergic receptor function, which may provide the molecular basis of peripheral nerves for the onset of mechanical hyperalgesia and the development of empathic pain. A recent study found that prazosin and propranolol, α2 receptor adrenergic antagonists, but not yohimbine, an α2 receptor adrenergic antagonist, completely eradicated hypersensitivity to mechanical pain in rats with CO. After removing noradrenergic innervations from the mPFC, however, researchers found that empathy no longer facilitated pain‐related behaviors in rodents. Adrenergic α1 or β receptor antagonists administered intra‐mPFC to CO rats eliminated the mechanical pain hypersensitivity associated with empathy. According to the findings, pain empathy in rodents is mediated by noradrenergic innervations of the mPFC via the α1 or β receptors.^87^

### 4.3 HPA axis

Stress stimulates parvocellular neurons in the hypothalamic PVN to produce corticotropin‐releasing hormone (CRH), arginine vasopressin (AVP), or both. CRH and AVP are released into the hypophyseal portal system and transported to the anterior pituitary, where CRH operates alone or in collaboration with AVP to stimulate the production of adrenocorticotrophic hormones from the pituitary corticotropes. Adrenocorticotrophic hormones stimulate glucocorticoid secretion by affecting the adrenal cortex. In both human and animal models, the empathic response (emotional contagion) is suppressed by activated HPA axis activity.^88^ Serum corticosterone levels were elevated, suggesting that the empathic response may involve a complex interplay between stressors, neuropeptides, and prefrontal cortical function.^89^ Negative feedback activity of glucocorticoids at many levels of the HPA axis reduces the stress response. Some centrally projecting CRH pathways are implicated in mediating behavioral reactions to stress (amygdala) and sympathetic activation (locus coeruleus).^90^ In the absence of oxytocin receptor density changes in neuronal networks involved in sympathetic behavior, researchers found that oxytocin administration normalizes emotional contagion, aggression, and behavioral stereotypies, resulting in increased compassionate behavior in mice. In addition, oxytocin administration resulted in a weaker and more prolonged neuroendocrine response to stress on the HPA axis in all mice.^91^ Specifically, changes in glucocorticoid and mineralocorticoid receptors were detected in brain regions associated with the regulation of the HPA axis, including the amygdala and hippocampus. Additionally, alterations in the opioid and oxytocin systems were identified in these brain regions. Worth noting is that the HPA axis is also implicated in the regulation of empathic behavior across species.^78^

### 4.4 Opioidergic system

The opioid system is among the most significant pain‐regulating systems ever identified. The opioid system is implicated in the self‐experience of pain. According to one study, it regulates the neural mechanism that initiates pain empathy, which is the perception of others' negative emotions.^92^ Numerous brain regions are responsible for regulating the opioid system, which governs both the sensory and affective dimensions of pain.^93^ The AI and anterior, middle cingulate cortex were reported to have higher opioid receptor concentrations and are activated by opioids.^94^ Considering its high‐density μ‐opioid receptors, the aMCC may specifically mediate the regulation of opioid drug activity, as seen in psychopharmacology experiments.^95^ fMRI reveals that upon observing pain in another individual, activation of brain regions implicated in opioid regulation—including the PFC and periaqueductal gray matter—indicates that the same brain regions responsible for opioid drug regulation are activated. This finding supports the notion that morphine influences empathy‐induced nociceptive changes. The use of both placebo analgesia and the opioid antagonist, naltrexone, in the study suggests that opioid agonists can reduce physical pain and inhibit cognitive and affective empathy in response to pain.^95^ Patients with chronic nonmedically prescribed opioids displayed lower levels of self‐related unpleasantness in response to others' pain than healthy controls.^96^ In one study, when opioid antagonists were used, the activity of the opioid system in the subject was observed, resulting in reduced discrimination of facial pain expressions.^97^ Because chronic morphine abuse reduces oxytocin secretion in the brain, long‐term administration of morphine may reduce empathic behavior by reducing the secretion of oxytocin. The role of oxytocin in pain empathy and affective pain processing in opioid‐dependent animals should be the subject of future research.^98^

### 4.5 5‐Hydroxytryptamine (5‐HT) signaling

Amygdala expression of 5‐HT3R has been demonstrated to be substantial.^99^ There are 15 subtypes of 5‐HT3R that play a significant role in chronic pain.^100^ Depending on the active site and receptor subtype, the amygdala regulates pain responses induced by 5‐HT. The amygdala regulates emotional processing and is closely linked to the 5‐HT system. Mice that live with a partner with a chronic contraction injury have increased conversion of serotonin in the amygdala, and administration of 5‐HT3R receptor blockers blunted the mouse writhing response.^101^ Coexisting in the same model of chronic pain, reduced and increased nociceptive responses in mice in both the amygdala 5‐HT3R and 5‐HT may modulate signals from sensory components in the mouse and regulate other affective aspects of pain. Amygdala and 5‐HT signaling—mainly mediated via 5‐HT3R—modulate pain hypersensitivity responses in mice cohoused with a lesion of the sciatic nerve.^26^ Present results corroborate this assumption, showing that 5‐HT3R blockade with systemic injections of ondansetron attenuated pain‐related behaviors. Intra‐amygdala injection of ondansetron completely blocked pain hypersensitivity induced by cohousing with a constriction‐injured partner.

## 5 EMPATHY AND CLINICAL PAIN

Evidence has shown that a history of chronic pain plays an important role in shaping empathy. Primary dysmenorrhea is not only a painful experience but also affects women's psychological and affective states. Research suggests that the gray matter volume of the brain structural network is associated with both pain intensity and scores of pain empathy in participants with primary dysmenorrhea compared with healthy controls. This indicated that long‐term pain may cause maladaptive brain structural plasticity, which may further affect psychological adjustment to bring patients more intense pain when they witness suffering and distress in others.^21^ Knee osteoarthritis is a degenerative type of arthritis characterized by chronic pain and widespread disability. The increased empathy of knee osteoarthritis patients towards pain indicates that past experiences may enhance empathy. Furthermore, the relationship between the severity of pain in knee osteoarthritis patients and personal pain related to empathy is mediated by psychological factors, including depression, anxiety, and catastrophic pain.^102^ Consistent with this finding, Fallon et al. found that fibromyalgia is characterized by widespread chronic pain, and patients not only show increased sensitivity to first‐hand experimental pain but also exhibit higher empathy scores.^103^ New evidence suggests that the common analgesic, paracetamol, may not only reduce physical pain but also alter the brain's response when perceiving pain from others and may also inhibit empathy processes.^104^ Considering the positive correlation between NE levels and empathetic behavior, as well as the negative correlation between dopamine levels and social behavior, it is possible that an enzyme that can regulate both dopamine and NE levels may in turn regulate an individual's ability to empathize and empathy‐related behavior. Dopamine beta‐hydroxylase is an enzyme that converts dopamine into NE. Furthermore, dopamine beta‐hydroxylase 1021 C/T is important as the genetic basis for empathy and in predicting individual differences in social and emotional processing.^105^ In a study on rats, the α1 subtype of adrenoceptors was also involved in pain empathy regulation; the rats were locally pre‐administered an α1 adrenoceptor antagonist, prazosin, through the subcutaneous administration pathway, which prevented the occurrence of empathetic mechanical hyperalgesia. However, an α2 or β adrenoceptor antagonist, such as yohimbine or propranolol, did not have this effect.^106^ Intranasal oxytocin can indirectly affect pain empathy ratings by reducing first‐hand pain sensitivity. Oxytocin has been shown to weaken neuronal activity in brain regions related to pain information processing and can indirectly downregulate pain empathy by reducing first‐hand pain sensitivity. Through a literature review, we found that primary dysmenorrhea, knee osteoarthritis, and fibromyalgia are diseases associated with pain empathy, while the drugs related to pain empathy in research mainly include acetaminophen, dopamine, NE, and oxytocin. In the future, more relevant clinical studies on empathy for pain will be conducted.

## 6 CONCLUSION

This study reviewed the neural regulatory mechanisms of pain empathy. Based on mirror neurons, pain empathy results from the combined action of brain regions such as the ACC, insula, PFC, and amygdala and regulatory substances such as oxytocin, the LC/NE system, the HPA axis, the opioid system, and 5‐HT. Understanding empathy for pain can help humans treat pain better in the future.

## AUTHOR CONTRIBUTIONS

Shuangshuang Liu and Jie Yuan conceived and designed the manuscript. Siwei Wang and Shuangshuang Liu contributed equally in writing the manuscript. YanYan, Qingxiang Mao, and Bangyong Qin edited the manuscript and modified the language of the manuscript as a final version. All authors read and approved the final version of the manuscript.

## CONFLICT OF INTEREST STATEMENT

Jie Yuan is a member of the Editorial Board of Ibrain and a coauthor of this article. To minimize bias, he was excluded from all editorial decision‐making related to the acceptance of this article for publication. The remaining authors declare no conflict of interest.

## ETHICS STATEMENT

Not applicable.

## Data Availability

Not applicable as no new data are generated in this review.
